# Ratiometric Temperature Sensing Using Highly Coupled Seven-Core Fibers

**DOI:** 10.3390/s23010484

**Published:** 2023-01-02

**Authors:** Daniel A. May-Arrioja, Miguel A. Fuentes-Fuentes, Iván Hernández-Romano, Rodolfo Martínez-Manuel, Natanael Cuando-Espitia

**Affiliations:** 1 Centro de Investigaciones en Óptica, Prol. Constitución 607, Fracc. Reserva Loma Bonita, Aguascalientes 20200, Mexico; 2 Coordinación de Matemáticas, Universidad Tecnológica de Aguascalientes, Blvd. Juan Pablo II 1302 Exhacienda la Cantera, Aguascalientes 20200, Mexico; 3 CONACyT-Electronics Department, Sede Palo Blanco, University of Guanajuato, Carr. Salamanca-Valle de Santiago Km 3.5 + 1.8, Salamanca 36885, México

**Keywords:** temperature sensor, multicore fiber, seven-core fiber, optical fiber sensors

## Abstract

In this paper, a ratiometric approach to sensing temperature variations is shown using specialty fiber optic devices. We analyzed the transmission response of cascaded segments of multicore fibers (MCFs), and dissimilar lengths were found to generate an adequate scheme for ratiometric operation. The perturbation of optical parameters in the MCFs translates to a rich spectral behavior in which some peaks increase their intensity while others decrease their intensity. Thus, by selecting opposite-behavior peaks, highly sensitive ratiometric measurements that provide robustness against spurious fluctuations can be performed. We implemented this approach using seven-core fiber (SCF) segments of 5.8 cm and 9.9 cm. To test the system’s response under controlled perturbations, we heated one of the segments from ambient temperature up to 150 °C. We observed defined peaks with opposite behavior as a function of temperature. Two pairs of peaks within the interrogation window were selected to perform ratiometric calculations. Ratiometric measurements exhibited sensitivities 6–14 times higher than single-wavelength measurements. A similar trend with enhanced sensitivity in both peak pairs was obtained. In contrast to conventional interferometric schemes, the proposed approach does not require expensive facilities or micrometric-resolution equipment. Moreover, our approach has the potential to be realized using commercial splicers, detectors, and filters.

## 1. Introduction

In recent years, an increasing interest has been demonstrated in implementing multicore fibers (MCFs) in fiber sensing systems [1,2,3]. Measurements and detection in deformation [4,5,6,7,8], refractive index [9,10,11,12], temperature [13,14,15,16,17,18,19,20,21], curvature [22,23,24], vibration [25], and polarization state [26] have been reported using MCF devices. MCFs have been described as revolutionary waveguides, as these fibers have allowed new alternatives for developing sensing systems whose performances cannot be easily achieved using conventional fiber optics [22]. Most of the reported fiber sensors based on MCFs are formed by simply splicing either one segment of a single mode fiber (SMF) or two segments of SMFs with an MCF segment having cores similar in diameter to the SMF. Such devices are known as SMF-MCF structures and SMF-MCF-SMF structures. In the SMF-MCF section, light traveling in the core of the SMF is coupled to the central core of the strongly coupled MCF, exciting then the so-called supermodes in the MCF [27]. The overlapping between the propagating modes in the different cores of the fiber generates a cyclical power transfer between them; in this sense, the transmission spectrum of these structures has the form of a periodical signal [28]. These reported sensor configurations are interrogated in transmission and also in reflection mode. In reflection mode, either in SMF-MCF or SMF-MCF-SMF structure, a mirror is implemented at the distal end of the fiber for guiding the light twice through the MCF structure, improving the narrowest band and visibility of the generated spectrum. Also, an MCF may have a symmetric or asymmetric arrangement of cores around the core at the geometrical center of the fiber. Both types of MCF have been exploited to generate novel sensor configurations. Typically, for sensing applications, the amplitude or the phase of the transmitted or reflected spectrum is monitored using an optical spectrum analyzer or photodetectors for signal investigation.

In terms of temperature monitoring using MCFs, the thermal expansion and thermo-optic coefficients for silica fiber change the length and refractive index of the MCF, respectively, as the MCF experiences changes in temperature. Therefore, each change in temperature in the SMF-MCF or SMF-MCF-SMF structure generates a shift in the transmission spectrum that is generally monitored using an optical spectrum analyzer (OSA). Recently in [14], we presented a temperature fiber sensor based on MCFs, capable of temperature monitoring using a pair of photodetectors. The system used two three-core fiber segments, with each segment in an independent arm, forming an SMF-MCF-SMF structure. Due to the asymmetrical position of the three cores, the MCF exhibited birefringence. Therefore, using similar MCF length and polarization controllers, the transmission spectra of the two sensing arms were phase-shifted by π radians from one another. Initiating the temperature monitoring at the quadrature point allowed a ratiometric monitoring scheme, as both sensing arms were exposed simultaneously to the changes in temperature. In ratiometric measurements, two points of measurement are implemented to obtain the change of the measurand by means of the ratio between them. Normally, these measurements are free of spurious fluctuations in the intensity of the optical source and losses in the optical sensor, clearly improving the sensing performance of the reported system [17,29,30,31,32,33]. The ratiometric measurement can be performed using two points of measurement, either having one affected by the measurand while the other unaffected or having both measurements affected in opposite directions.

Unlike our previous work [17], we exploit the versatility of MCFs for tuning each SMF-MCF-SMF device’s spatial frequency and selecting the fiber system’s spectral response for ratiometric operation. The selection is based on the concatenated response of two SMF-MCF-SMF segments of different spatial frequencies, which also deviates from the parallel fiber setup used previously. For fibers with fixed geometric and material parameters, the spectral response of an SMF-MCF-SMF device is a function of the length of the MCF segment. Thus, the spectral response of two SMF-MCF-SMF devices connected in cascade is a function of the product of the individual responses. Furthermore, in the present approach, only one MCF segment is exposed to the change in temperature, while the other is isolated and used as a reference. A theoretical model and experimental results of the proposed ratiometric temperature sensor are presented.

## 2. Simulations

The transmission of an SMF-MCF-SMF structure can be modeled within the coupled-mode theory [21]. In a centrosymmetric MCF such as a seven-core fiber (SCF), one can assume that the light from the inlet SMF couples to the central core of the SCF. We can also assume that the light transmitted to the outlet SMF corresponds to the light present in the central core of the SCF at the outlet SCF-SMF interface. Previous works have demonstrated a good correlation between this approach and experimental results [12,21]. Among the advantages of using SCF as a sensing element are the ease of integration with fiber optics technology, reduced size and mass, and an interferometric-like behavior characterized by a periodic spectral response. This periodic response is highly adequate in optical sensing as the variation in intensity, phase, and frequency can be easily monitored. The SCF used in this work consisted of a central core and six external cores of the same dimensions and refractive indices. A detailed description of this type of fiber can be found in references [18,19,20]. Briefly, each core can be seen as an individual waveguide and according to coupled-mode theory, the energy from the central core couples to the external cores as a function of wavelength and transmitted length. More precisely, the normalized transmitted intensity *I* in an SMF-SCF-SMF is a function of the length *L* of the SCF and the coupling coefficient *κ* as follows [21]:

(1) $$I=\frac{1}{7}\left[4+3\cos\left(2\sqrt{7}L\kappa \right)\right].$$

In general, *κ* is a function of wavelength and depends on the fiber’s geometric construction and refractive indices [34]. An SMF-SCF-SMF exhibits a sinusoidal behavior in wavelength for a fixed length *L*. According to Equation (1), the spatial frequency of these structures is directly proportional to the product *Lκ*. In contrast to our previous work [17], in which we studied the reflected signals from SMF-SCF-SMF devices, here we explore the behavior of transmitted signals of SMF-SCF-SMF devices in a cascaded configuration. Transmission measurements in cascade configuration do not require metallic coatings or fiber couplers to acquire the corresponding signal. Moreover, it has been demonstrated that the envelope of transmitted/reflected and cascaded/parallel signals from two SMF-SCF-SMF devices contain similar frequency components [21].

For two SMF-SCF-SMF devices of different lengths, the normalized transmitted intensity when the devices are connected in cascade (*I_c_*) is [21]:

(2) $$I_{c}=\left(A+B\cos(2\sqrt{7}L_{1}\kappa )\right)\left(C+D\cos(2\sqrt{7}L_{2}\kappa )\right).$$

**Figure 1:**
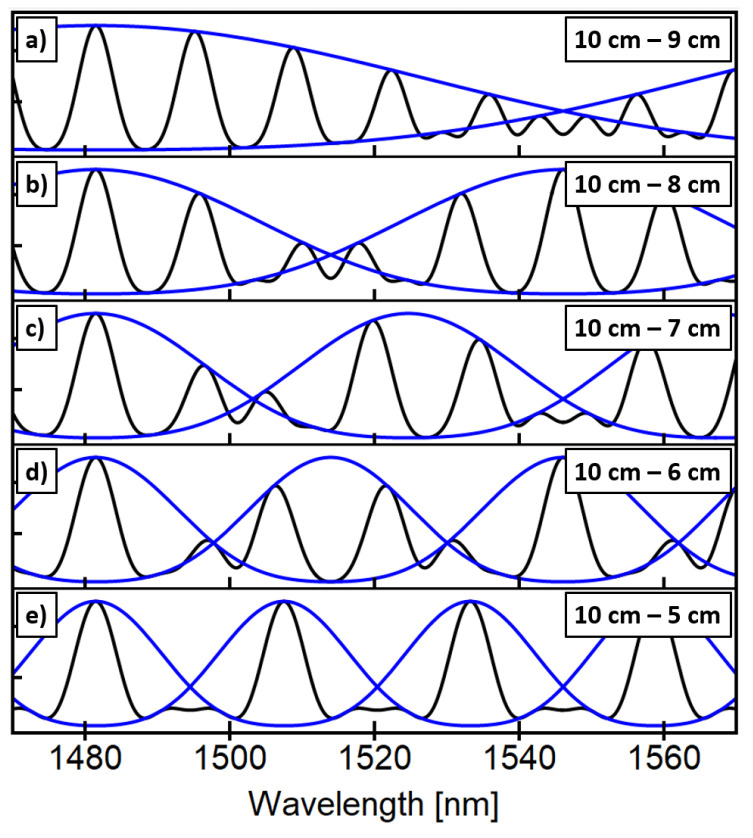
Simulated transmitted spectral response of two cascaded SMF-SCF-SMF with SCF fiber length combinations of (**a**) 10 cm–9 cm, (**b**) 10 cm–8 cm, (**c**) 10 cm–7 cm, (**d**) 10 cm–6 cm, and (**e**) 10 cm–5 cm. The blue curve corresponds to each case’s best fitting envelope of the transmitted spectrum (black curve).

Basically, Equation (2) is the product of two individual SMF-SCF-SMF devices. The constants *A–D* in Equation (2) are related to intrinsic losses, imperfect splices, and fiber optic devices which can be experimentally determined. Moreover, the resulting spectrum exhibits an envelope which is a function of the difference between their trigonometric arguments. To study this behavior, we have simulated the response of two cascaded SMF-SCF-SMF devices with different SCF lengths and showed the resulting spectra in Figure 1. The fiber parameters used in the simulations were taken from reference [21]. Spectra of cascaded SCF devices with 10 cm–9 cm, 10 cm–8 cm, 10 cm–7 cm, 10 cm–6 cm, and 10 cm–5 cm are plotted in Figure 1a–e, respectively.

Under these conditions, two extreme scenarios can be observed. First, when the spatial frequencies of the cosine terms are very similar (i.e., similar SCF lengths), a low-frequency envelope modulates the output spectrum (see Figure 1a,b). This effect is known as the Vernier effect and has been recently used to enhance the sensitivity of fiber optic sensors [21,35,36]. On the other hand, as depicted in Figure 1e, when one of the SCF lengths doubles the SCF length of the remaining device (10 cm and 5 cm), a periodic train of peaks is obtained. In this harmonic case, the transmitted spectrum’s peaks coincide with the envelope’s maximum, and the contrast between transmitted peaks is zero as peaks exhibit the same amplitude. Nevertheless, an even more interesting situation occurs when the spatial frequencies of the SCFs devices become less similar. As shown in Figure 1c,d signal and envelope maxima do not coincide, which translates into a sharper contrast between adjacent peaks in the output transmission spectrum. If we take a closer look at the peaks centered around 1500 nm, we can observe that as we change the SCF length from 7 cm to 6 cm, the peaks change from maximum to minimum and vice versa. This behavior is an interesting feature because the net effect of changing the SCF length, with respect to a fixed 10 cm SCF segment, is a change in frequency which translates into peak intensity changes. Moreover, this is an adequate situation for ratiometric measurements since it benefits from spectra with high-contrasted peaks and intensities moving in the opposite direction for different peaks, as shown in Figure 1c,d.

**Figure 2:**
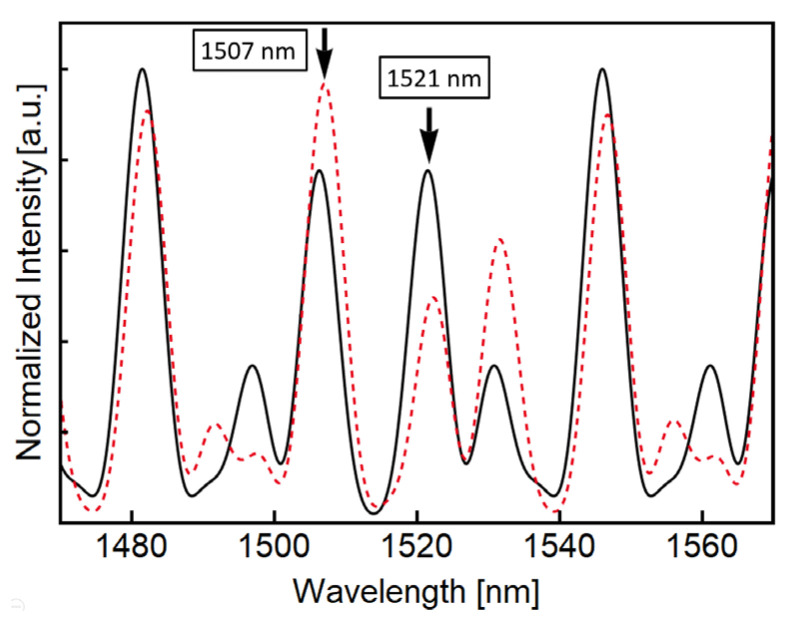
Simulated transmitted spectral response of two SMF-SCF-SMF devices of 10 and 6 cm. The black solid curve corresponds to the response at room temperature using Equation (2). The red dotted curve corresponds to the same devices when the temperature in the SMF-SCF-SMF device of 6cm is increased by 100 °C. The black arrows indicate the ratiometric behavior. The intensity increases with temperature for some peaks, while the opposite behavior is observed for other peaks.

When this cascaded configuration is used for temperature measurements, the SCF lengths are fixed, and the temperature is changed only in one of the SCF segments. Under this scenario, a disturbance in the SCF parameters will mainly shift the envelope of the output spectrum, and we expect similar changes in the peak’s intensity with some of them going in opposite directions. With this in mind, we selected SCFs devices of 10 cm and 6 cm to numerically study the response of the transmission spectrum subjected to temperature variations. Figure 2 shows the simulated transmission response of the selected SMF-SCF-SMF devices when both SCF segments are at room temperature (solid black line) and when the 6 cm segment is heated to 125 °C while maintaining the other segment at room temperature (red dashed line). The solid curves shown in Figure 2 are constructed using Equation (2) and assuming a room temperature of 25 °C. The red dotted lines shown in Figure 2 correspond to the same devices’ response but with an increment of 100 °C in the SCF segment of 6 cm while maintaining room temperature in the rest of the experimental setup. In order to simulate the temperature increment, the thermo-optic coefficients of cladding and core (9.5 × 10^−6^/°C and 9.75 × 10^−6^/°C, respectively) were used as in [37]. Although thermal expansion also affects the spectral response of SCFs, the thermal expansion for fibers has been calculated to be around two orders of magnitude smaller than the thermo-optic coefficient [38,39]. Thus, a valid approximation is to assume that the effects of temperature variation in fiber optic sensors are related mainly to the thermo-optic coefficient [40].

The effect of temperature on individual SMF-SCF-SMF devices has been studied previously [20]. In this reference, a wavelength shift of 29 pm/°C in the transmitted signals was reported, leaving the main cosine structure of the signals unaffected. However, the dotted curves of Figure 2 are not a shifted version of their solid counterparts. The dotted curves of Figure 2 show a similar structure of peaks and valleys located around the same wavelengths as their solid counterparts. For some wavelengths, the dotted curve of Figure 2 exhibits an increase in intensity. In contrast, the dotted curve decreases its intensity for some other wavelengths compared to the solid curve. For instance, compare the peaks around 1507 nm and 1521 nm in Figure 2, indicated with black arrows. For room temperature, both peaks present similar intensities. However, when the temperature increment is simulated in the SCF segment of 6 cm, the peak around 1507 nm increases its intensity while the peak at 1521 nm decreases in intensity.

The temperature perturbation in these configurations induces an increase and decrease of intensity in defined peaks simultaneously, allowing for tracking the temperature changes by the ratio of two defined peaks rather than by the absolute intensity. In general, for measurements based on the absolute intensity of a given peak, any variation in the power of the light source may lead to erroneous readings. However, the ratio of two peaks is unaffected by the variation in light source power, leading to more robust readings. Moreover, according to these simulations, the separation between adjacent peaks (~14 nm) and their corresponding spectral width (FWHM~6 nm) allows for the implementation of interrogation schemes of individual peaks using commercial fiber devices.

## 3. Experimental Results

**Figure 3:**
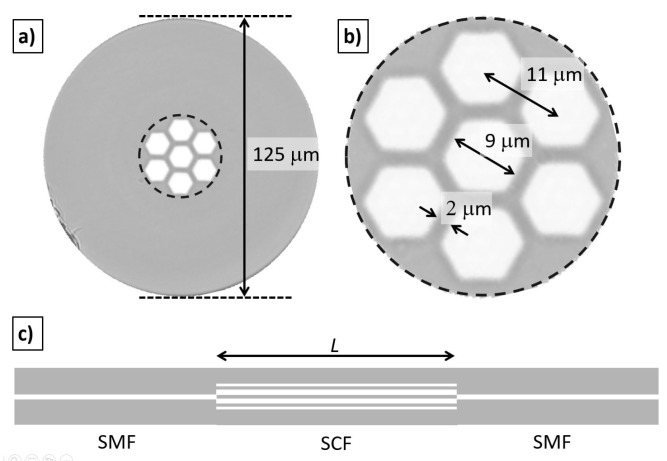
Transversal section of the SCF used in this work. (**a**) The fiber diameter is 125 µm, and the hexagonal cores are arranged in a honeycomb-like distribution. (**b**) Detail of the fiber cores showing a separation of 11 µm between center cores and a core size of 9 µm measured between opposite facets. (**c**) Schematic of the transversal section of the fabricated SMF-SCF-SMF devices.

Based on the results of the previous section, we fabricated a ratiometric temperature sensor based on two SMF-SCF-SMF devices. An image of the SCF used to fabricate the ratiometric sensor is shown in Figure 3. The cores of the SCF have a hexagonal shape, with a central core and six external cores, which are symmetrically positioned with respect to the faces of the hexagonal central core. The refractive index (RI) of the cores and cladding are 1.450 and 1.444, respectively. The external cladding has a diameter of 125 µm which facilitates the splicing with standard SMF (see Figure 3a). As shown in the zoomed region of Figure 3b, the center-to-center separation between the cores is 11 µm, and the size of the core measured between opposite facets is 9 µm which provides a separation between the cores of 2 µm. This SCF was supplied by the Microstructured Fiber and Devices Group at CREOL, The College of Optics and Photonics, University of Central Florida.

The fabrication of the sensors is rather simple since we only need to splice a section of SCF between two segments of SMF, as shown in Figure 3c. Based on our simulations, two sections of SCF fiber with different lengths are needed to observe the ratiometric behavior. The fabrication of the sensors is achieved by first splicing an input SMF to a segment of SCF. Afterward, the splicing point is placed at a specific distance with respect to the knife of the cleaver, and with the aid of two translation stages, the SCF lengths are fixed and cleaved. Finally, the cleaved end of the SCF is spliced to the output SMF. Sensors with SCF lengths of 5.8 cm (*L*_1_) and 9.9 cm (*L*_2_) were obtained, which are quite close to the simulated lengths of 6 cm and 10 cm. We should highlight that we did not assemble a highly accurate cleaving station because, according to our simulations, such small length differences do not significantly modify the expected spectral response of the combined sensors.

**Figure 4:**
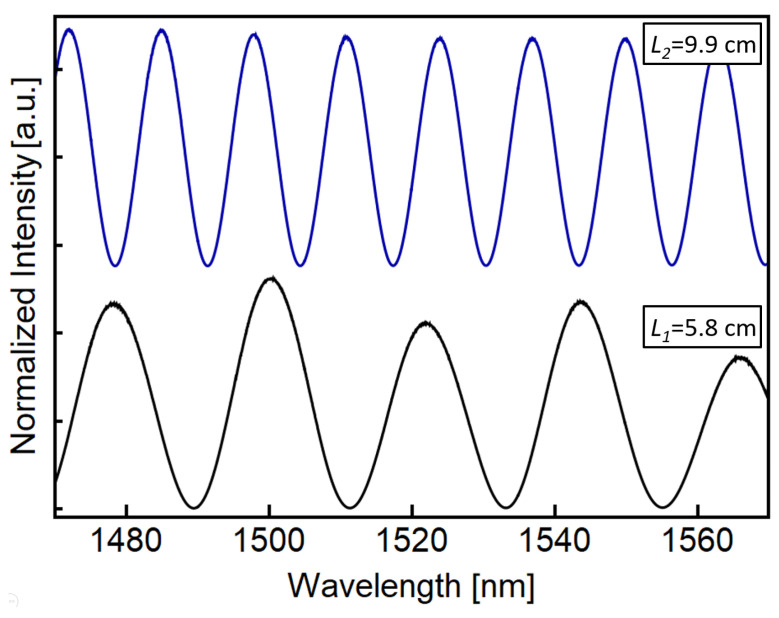
Individual transmitted spectral response of the SMF-SCF-SMF devices. Each SCF length is indicated with an increment in the wavelength frequency as we increase the SCF length.

The experimental spectral response of both SCF lengths is shown in Figure 4. As expected, by increasing the SCF length, the frequency of the transmitted oscillations is also increased. We can also correlate the experimental frequency response of each SCF with its actual length, and the agreement is quite good. It is worth mentioning that the central core of the SCF allows for simple alignment to an SMF using the cladding splicing mode of the fusion splicer. Nevertheless, every splice always has some uncertainty, which can be related to slight variations in the cladding diameter. Although a small insertion loss difference can be observed for both SCF lengths, as will be shown later, this does not affect the performance of the combined responses.

**Figure 5:**
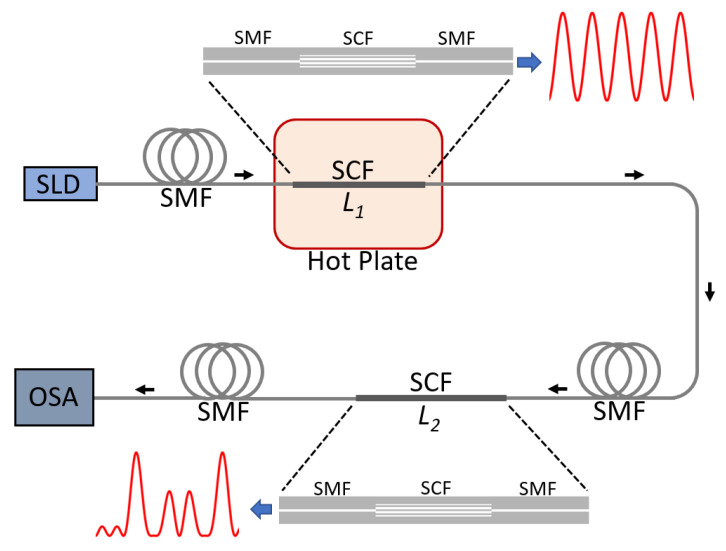
Experimental setup for temperature measurements using the concatenated SMF-SCF-SMF devices. SLD: superluminescent diode, OSA: optical spectrum analyzer, SMF: single-mode fiber, SCF: seven-core fiber. Red curves indicate representative spectra after each SMF-SCF-SMF device. Black arrows indicate the direction of light inside the fiber sensor.

The experimental setup for temperature measurements consists of a superluminescent diode (SLD, Thorlabs, SLD1550S-A2, bandwidth 150 nm) as the broadband source to interrogate the sensing device and an OSA (Anritsu MS9740A) to acquire the transmitted spectrum. The temperature of the sensing SCF section was changed using a ceramic hotplate (IKAC-MAG HS7). Temperature changes were monitored using a type K thermocouple located at the center of the hotplate, and data were recorded using a data acquisition card (DAQ, National Instruments, USB-6501). A small aluminum box was machined and placed on top of the hotplate to minimize temperature fluctuations due to ambient airflow. Care was taken to register the spectral measurements only after the temperature readings in the thermocouple showed a stable value. A schematic representation of the experimental setup is depicted in Figure 5.

As shown in Figure 5, we spliced both SCF segments in series, with the SLD connected to the shorter SCF (*L*_1_) and the OSA connected to the output of the longer SCF (*L*_2_). The SMF segment between *L*_1_ and *L*_2_ was on the order of 2 m. This segment and *L*_2_ were fixed and in contact with the optical table during all the experiments to minimize the heat diffusion from *L*_1_ to *L*_2_. The spectral response as a function of temperature was acquired by heating the sensing *L*_1_ segment. The temperature was changed from 24 °C to 124 °C in steps of 25 °C, while the reference *L*_2_ segment was kept at room temperature (24 °C).

**Figure 6:**
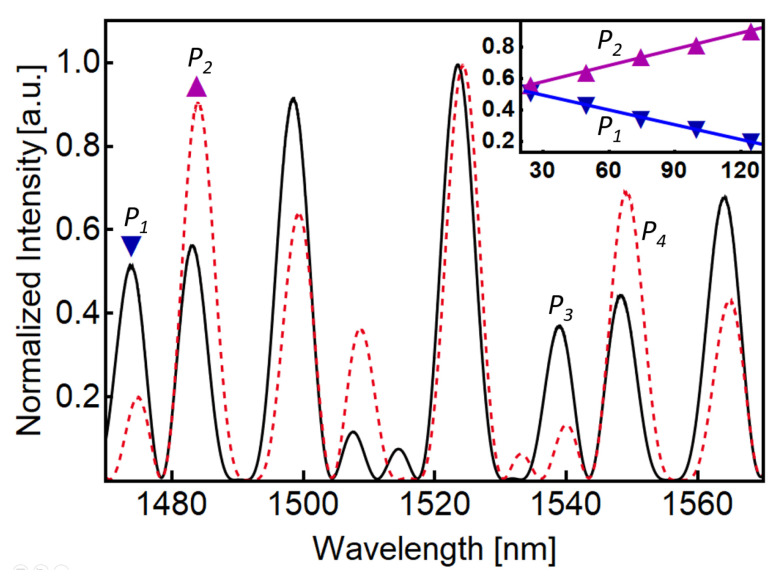
Experimental spectral response of the concatenated SMF-SCF-SMF devices when *L*_1_ is at room temperature (black curve) and heated at 124 °C (dashed red curve). The inset shows the peak intensity of *P*_1_ and *P*_2_ as a function of temperature.

The spectral behavior of the constructed sensor at ambient temperature is shown in Figure 6 as a solid black curve. The dotted red curve in Figure 6 represents the recorded spectrum of the sensor when *L*_1_ is heated to 124 °C. We only show minimum and maximum temperature values to facilitate observation of the peak intensity variation as a function of the applied temperature. In general, Figure 6 resembles the response of the simulated device shown in Figure 2, with peaks of different intensities in which the temperature perturbation decreases the intensity of some peaks while increasing the intensity of adjacent peaks. To implement ratiometric measurements, we have selected two pairs of neighboring peaks and studied their peak intensity as a function of the temperature. We selected the peaks at 1473, 1483, 1538, and 1549 nm, and we labeled those peaks as *P*_1_, *P*_2_, *P*_3_, and *P*_4_ in Figure 6. As shown in Figure 6, the first pair of selected peaks, *P*_1_ and *P*_2_, exhibit a similar intensity at room temperature. From the nine defined peaks observed in the solid black curve of Figure 6, the peaks at 1538 nm and 1549 nm (*P*_3_ and *P*_4_, respectively) also show a similar intensity. Moreover, we can observe that as the temperature increases, the intensity of *P*_2_ and *P*_4_ increases while the intensity of *P*_1_ and *P*_3_ decreases. This behavior can be easily observed by plotting the peak intensity of each peak as a function of the applied temperature, as shown in the inset of Figure 6. In Figure 6’s inset, *P*_1_ and *P*_2_ peak intensities are plotted as a function of the applied temperature to *L*_1_. The inset shows the intensity of *P*_1_ as inverted blue triangles and *P*_2_ as upright purple triangles. In addition, solid magenta and blue lines show linear fittings to the corresponding experimental data. It is worth mentioning that the response is not only highly linear, but this opposite behavior is adequate to perform ratiometric measurements. Moreover, by selecting the decreasing peak as the denominator in the ratiometric measurement, the intensity ratio is highly enhanced as the temperature increases. A similar linear and opposite behavior was observed analyzing *P*_3_ and *P*_4_ (plot not shown for clarity).

**Figure 7:**
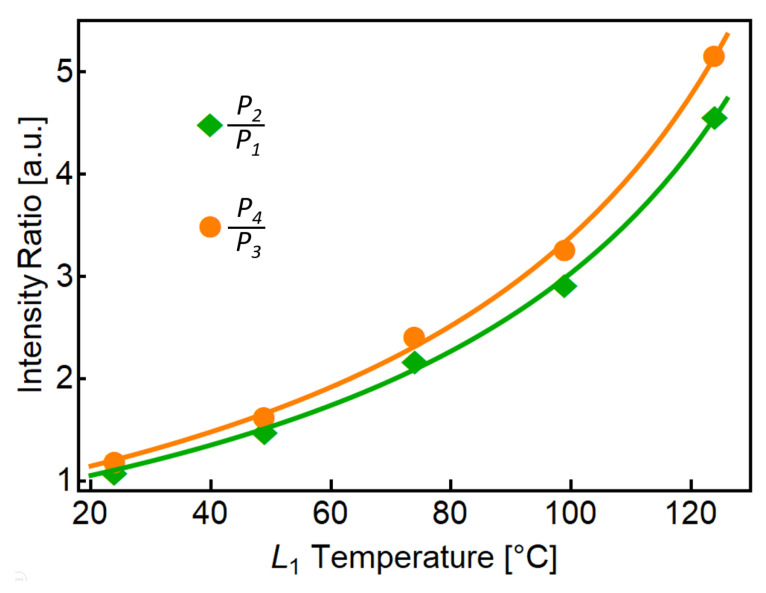
Intensity ratio as a function of applied temperature with solid green and solid orange curves corresponding to the ratios of peaks *P*_2_/*P*_1_ and *P*_4_/*P*_3_, respectively.

The ratiometric operation in both pairs of peaks was obtained using the experimental data, and the results are shown in Figure 7. In particular, Figure 7 shows the calculated ratios *P*_2_/*P*_1_ (green diamonds) and *P*_4_/*P*_3_ (orange circles) as a function of the recorded temperature in *L*_1_. We observed a non-linear behavior in the ratiometric measurements, as expected from the quotient of two generalized lines with non-zero intercepts. To display this behavior, we have also plotted in Figure 7 the ratio of the calculated fittings from Figure 6. These fittings are depicted in Figure 7 as solid green and solid orange curves corresponding to *P*_2_/*P*_1_ and *P*_4_/*P*_3_ ratios, respectively. Notice that we have selected the denominator to emphasize the ratio as the temperature increases. Notice also that the vertical axis labeled as intensity ratio measures the proportion between peaks and does not require previous normalization. In other words, this measure is normalized to one of the selected peaks and does not depend on the power fluctuations of the interrogation source.

Interestingly, the intensity ratio is higher for the second pair of adjacent peaks (orange circles), which is not evident from the spectra of Figure 6. This result is related to the small values of *P*_3_, which enhances the ratio *P*_4_/*P*_3_, and because *P*_3_ is smaller than *P*_1_ for the studied temperature range (compare *P*_1_ and *P*_3_ in the solid black curve and dotted red curve of Figure 6). The calculated fittings have a good agreement with the experimental data and show the desired/expected accelerated increase in intensity ratio as the temperature increases.

## 4. Discussion and Conclusions

The spectral response of concatenated SMF-SCF-SMF devices was studied in this work. The sinusoidal spectral response obtained by individual SMF-SCF-SMF devices provides an ideal ground for the realization of novel sensing mechanisms. For example, we could operate within the optical Vernier regime by using similar SCF lengths in the concatenated SMF-SCF-SMF devices [21]. By changing the SCF length in one SMF-SCF-SMF to avoid Vernier operation and harmonic operation, i.e., exact multiples between SCF lengths, we reach a regime where a rich spectral behavior is obtained. In this regime, several peaks are observed within the operational spectral window, exhibiting different properties when the SCF optical properties are modified. For instance, when the SCF is disturbed, some peaks will increase their intensity, others will be reduced, and some will not be altered. Although we took advantage of peaks changing in opposite directions in our proposal, as required for ratiometric operation, other applications could benefit from peaks with different behaviors. Single-wavelength measurements correspond to a slope of roughly ±0.3 in normalized intensity units within a range of 100 °C (±0.003 a.u./°C), as shown in the inset of Figure 6. In contrast, Figure 7 shows an increment of more than three times in the same normalized intensity units within the same temperature range for both ratiometric measurements. A linear fitting using the first three experimental green points in Figure 7 (temperature range from 24 to 74 °C) leads to a slope of 0.021 a.u./°C, while the slope of a linear fitting using the last three green points (temperature range from 74 to 124 °C) results in a slope of 0.47 a.u./°C. This comparison indicates an enhancement in sensitivity of 6–14 times higher than single-wavelength measurements. It is important to note that the ratiometric calculations result in a non-linear curve because the single-wavelength measurements have a different slope. Therefore, ratiometric results are highly stable and reproducible since they originate from highly linear experimental results. We should also emphasize that the potential temperature operational range can be significantly large since the SCF is made from silica. Ideally, the temperature can be as low as −170 °C [41] and as large as 1000 °C [20]. In our experiments, the applied temperature was limited by the available equipment.

We should also emphasize that the fabrication of the SMF-SCF-SMF is relatively simple and does not require any special cleaving or splicing procedures. The SCF lengths were cleaved without using highly accurate stages, and the splicing between SMF and SCF used a standard splicing procedure in our Fujikura 60S splicer. Our fabrication process is straightforward if we compare it to very complex fiber structures that have been reported to achieve interferometric devices that can provide a sinusoidal spectral response [42,43]. This is a critical issue for a potential commercial application where simple fabrication processes are highly convenient. Note that in these experiments, we induced temperature changes to one of the SMF-SCF-SMF devices while the other was isolated by physical separation and contact maintained with an optical table. In potential applications, this insulation can be implemented by fiber encapsulation and also by physical separation of the two devices.

Additionally, we should also note that for clarity purposes, the transmitted spectral responses are presented using an OSA because it allows us to show the rich peak spectral response of the concatenated devices. However, after adequate peaks have been identified for ratiometric operation, we could use spectral bandpass filters to measure only the signal from those two peaks. In this sense, spectral measurements are not required, and the use of photodetectors could significantly reduce the cost of the sensing system. Finally, it is also important to note that ratiometric measurements are free of spurious fluctuations generated by the optical source and losses in the optical sensor. The advantages, as mentioned earlier, make our ratiometric sensor highly robust and versatile during its fabrication and operation.

In conclusion, a versatile ratiometric sensing scheme is presented based on highly coupled multicore fibers. The present approach offers significant advantages for potential commercial applications, such as ease of implementation, high sensitivity, and robustness over source fluctuation. Finally, we believe this report may allow the design and fabrication of fiber optic sensors for various physical parameters based on the proposed approach.

## Data Availability

Data is contained within the article and more details are available on request from the corresponding author.
